# The use of electronic alerts in primary care computer systems to identify the over-prescription of short-acting beta_2_-agonists in people with asthma: a protocol for a systematic review

**DOI:** 10.1038/s41533-017-0033-y

**Published:** 2017-04-26

**Authors:** Shauna McKibben, Andy Bush, Mike Thomas, Chris Griffiths

**Affiliations:** 1 Asthma UK Centre for Applied Research, Queen Mary University London, London, UK; 2 Asthma UK Centre for Applied Research, Imperial College London, London, UK; 3 Asthma UK Centre for Applied Research, University of Southampton, Southampton, UK

## Background

Asthma is a heterogeneous disease, usually characterised by (a) chronic airway inflammation with variable symptoms of wheeze, shortness of breath, chest tightness and/or cough, and (b) variable expiratory airflow limitation.^1^ Despite increasing evidence-based guidelines for asthma gaps between recommended care and current practice remain.^2, 3^ Frequent and increasing use of short acting beta_2_-agonists (SABA) or reliever therapy is a marker for poor asthma control and increased risk of asthma attacks,^4^ with control defined as the degree to which the manifestations of asthma are minimised by treatment.^5^ Asthma control can be assessed by reviewing both current symptoms and risk factors (modifiable or non-modifiable) of future asthma attacks.^1, 6^ Poor asthma control and risk of asthma attacks can be determined in part by SABA use,^7–12^ with high or increasing SABA use a potentially modifiable risk factor for asthma attacks^9, 11, 13, 14^ and asthma related death.^7, 8, 15^ Poor asthma control is commonly due to suboptimal asthma management and can result not only in loss of school and workdays at a high cost for countries^16–18^ but also in unnecessary morbidity and even mortality.^3, 19^ The National Review of Asthma Deaths identified that 39% of people who died from asthma had been prescribed more than 12 SABA inhalers in the year before death and 4% had been prescribed more than 50 SABA inhalers in the year before death.^15^ Recent figures show that asthma deaths are at the highest level for a decade with a 17% increase in the number of asthma related deaths from 2014 to 2015 in England and Wales.^20^

Computer decision support systems (CDSSs) are increasingly being used to improve the prevention and management of chronic conditions such as asthma.^21, 22^ CDSSs include electronic alerts and reminders that use patient-specific information and clinical data to help healthcare providers make decisions that enhance patient care.^21, 23^ Whilst CDSSs have the potential to improve prescribing efficiency for healthcare professionals^24–27^ overall effectiveness in clinical practice is unclear.^28^ Recommendations have called for the electronic surveillance of prescription refill frequency in primary care to alert clinicians to patients being prescribed excessive quantities of SABA^15^; however it is unclear to what extent alerts have been used in the management of SABA prescribing and what impact, if any, they have on patient outcomes.

## Aims

We aim to identify and critically appraise studies that have used electronic alerts to identify people with asthma being prescribed excessive SABA in primary care.

Specific objectives are as follows:

1. Evaluate the effectiveness of electronic alerts within CDSSs to identify people with asthma being prescribed excessive SABA in primary care.
2. Determine the features of electronic surveillance systems that have the potential to improve process outcomes for healthcare providers and clinical outcomes for people with asthma.

## Discussion and conclusion

CDSS interventions can potentially increase adherence to evidence-based medical knowledge, reduce unnecessary variation in clinical practice and improve clinical decision-making processes^29, 30^ particularly in the prevention and management of chronic conditions.^21^ Studies addressing the use of CDSSs in the care of people with asthma have shown varying results. One study reported that CDSSs had little effect on clinical and process outcomes for asthma due to low clinician use^22^ whilst another reported that CDSSs can improve chronic disease processes and outcomes particularly in the support of asthma self-management.^21^ Alerts represent an important category of decision support to clinicians, often having a substantial effect on prescribing behaviour.^31^ However few studies have assessed the impact of computerised alerts on clinical or health service management outcomes.^31^ Current recommendations include the use of alerts within general practice computer software to identify patients with asthma being prescribed excessive quantities of SABA.^15, 32^ A thorough synthesis of the evidence is required to: (i) determine the extent to which electronic alerts to identify people with asthma being prescribed excessive SABA in primary care can improve asthma management and patient outcomes; (ii) clarify the design and implementation of CDSSs alerts to improve asthma prescribing decisions for clinicians.

## Methods

### Study eligibility criteria

#### Types of studies

We will include all types of randomised controlled trials in which patients have been treated by clinical teams informed by an electronic SABA prescribing alert compared with usual care. As a surrogate measure of prescribing, studies using dispensing data will be included. We will exclude non-randomised trial designs (quasi-experimental, observational studies); study protocols; paper-based tools (e.g., flow charts and non-electronic clinical pathway tools); CDSS alerts used for conditions that are not asthma, e.g., COPD or other respiratory conditions; CDSS alerts used in secondary or tertiary care.

#### Types of participants

We will include studies involving healthcare professionals and non-clinical staff in primary care who provide care to adults and/or children with a physician coded asthma diagnosis.

#### Types of intervention

We will include studies which used CDSS based alerts initiated by the excessive prescribing of SABA for people with asthma. Definitions of excessive prescribing will be analysed on an individual study basis.

#### Types of outcome measures

The primary outcome will be study-defined SABA over-prescription. Secondary outcomes will be SABA prescribing, inhaled corticosteroid (ICS) prescribing alone or with a long-acting beta_2_-agonist, the ratio of ICS-SABA prescribed, asthma reviews, study-defined asthma exacerbations, study-defined asthma exacerbations requiring oral steroids, unscheduled consultations for asthma (including general practice visits, emergency department visits and hospitalisations for asthma) and study-defined asthma control assessment.

### Search strategy

We will search the international electronic databases: MEDLINE (Ovid), EMBASE (Ovid), CINAHL (Cumulative Index to Nursing and Allied Health Literature), SCOPUS (Elsevier) and Cochrane Library (Wiley). Additional studies will be retrieved by searching the references of eligible papers. Unpublished and in-progress studies will be identified by searching online trial registries; ISRCTN registry and ClinicalTrials.gov. All databases will be searched from 1990 to July 2016. No language restrictions will be imposed; translations will be sought where possible. Supplementary Appendix 1 presents details of our search strategy, which was developed for MEDLINE and will be adapted in searching other databases.

#### Screening of retrieved literature

The titles and abstracts of all papers retrieved from the databases will be checked independently by two reviewers against the criteria of the study. The full texts of papers that are potentially eligible will be retrieved and further assessed for inclusion independently by two reviewers. Discrepancies in the screening processes between the two reviewers will be resolved by consensus, and disagreements will be arbitrated by a third reviewer.

### Data extraction

A customised data collection form will be used by two reviewers, independently, to extract relevant study data from full-text papers selected for inclusion. The form will be piloted and refined before being applied to full-text reports. Included papers will be discussed by the two reviewers after data extraction, and disagreements will be arbitrated by a third reviewer. Where necessary, clarification and additional data will be sought from study authors. Key findings from each included study will be summarised and tabulated.

### Quality assessment

We will assess the risk of bias in each trial using the seven-criteria approach described in section eight of the Cochrane Handbook for Systematic Reviews of Interventions.^33^ Overall, each study will be rated as follows: A: low risk of bias—no bias found; B: moderate risk of bias—one criterion for risk of bias; C: high risk of bias—more than one criterion for risk of bias.

### Data synthesis

Narrative synthesis of heterogeneous process outcomes (prescribing and asthma reviews) and clinical outcomes (exacerbations, unscheduled consultations and asthma control) will be conducted. Data will be presented in tabular form. Where possible, meta-analysis will be performed on process and clinical variables of interest, specifically: study-defined SABA over-prescription, study-defined asthma exacerbations and study-defined asthma control.

Heterogeneity will be assessed using the I-squared statistic. Where possible, subgroup analyses will be performed on age categories as defined by BTS/SIGN Guidelines; less than 5 years, aged 5–12 years and greater than 12 years of age.^4^

### Registration and reporting

This study is registered with PROSPERO, the University of York Centre for Reviews and Dissemination International prospective register of systematic reviews (CRD42016035633).

We will report according to the PRISMA guidelines for reporting systematic reviews.^34^

## Electronic supplementary material

Appendix 1
