# Supplementary material for: The use of electronic alerts in primary care computer systems to identify the over-prescription of short-acting beta2-agonists in people with asthma: a protocol for a systematic review
Source: NPJ Prim Care Respir Med. 2017 Apr 26;27:30. doi: 10.1038/s41533-017-0033-y (PMC5435095; doi:10.1038/s41533-017-0033-y)
Supplement: Supplementary file 1 — Appendix 1 [file 41533_2017_33_MOESM1_ESM.docx]

**Appendix 1: Search strategy for Medline (Ovid)**

1. exp ASTHMA/
2. asthma.ti,ab
3. 1 OR 2
4. exp DECISION SUPPORT SYSTEMS, CLINICAL/
5. exp DECISION SUPPORT SYSTEMS, MANAGEMENT/
6. exp DECISION SUPPORT TECHNIQUES/
7. exp DECISION MAKING, COMPUTER-ASSISTED/
8. exp MEDICAL RECORDS SYSTEMS, COMPUTERIZED/
9. "decision support".ti,ab
10. "expert system$".ti,ab
11. cdss.ti,ab
12. 4 OR 5 OR 6 OR 7 OR 8 OR 9 OR 10 OR 11
13. 3 AND 12
